# Effects of Tissue Flossing on the Healthy and Impaired Musculoskeletal System: A Scoping Review

**DOI:** 10.3389/fphys.2021.666129

**Published:** 2021-05-21

**Authors:** Andreas Konrad, Richard Močnik, Masatoshi Nakamura

**Affiliations:** ^1^Institute of Human Movement Science, Sport and Health, Graz University, Graz, Austria; ^2^Institute for Human Movement and Medical Sciences, Niigata University of Health and Welfare, Niigata, Japan

**Keywords:** occlusion, blood flow restriction, voodoo band, flexibility, strength, recovery

## Abstract

There is a belief that tissue flossing can improve the range of motion or performance, speed up recovery, and decrease the pain caused by various diseases or injuries. As a result, many therapists, patients, and athletes are now using this technique. Consequently, in the last 5 years, a number of studies have addressed these assumptions. The purpose of this scoping review is to introduce the application of a floss band and to summarize the existing evidence for the effect of floss band treatment on the range of motion, performance, recovery, and pain (due to disease or injuries). A further goal is to suggest what needs to be addressed in future studies. The online search was performed in PubMed, Scopus, and Web of Science databases. Any studies dealing with the effects of a floss band treatment on the range of motion, performance, recovery, or pain parameters in any population (e.g., patients, athletes) were included in this review. Twenty-four studies met the inclusion criteria, with a total of 513 participants. The included studies revealed that there is evidence that a single floss band treatment is able to increase the range of motion of the related joint and can positively affect jumping and strength performance. However, these findings show only small to moderate effect sizes. Although not yet clearly understood, a possible mechanism for such changes in the range of motion or performance is likely due to changed neuromuscular function, rather than changed mechanical properties, of the muscle (e.g., stiffness). All in all, there is a need to conduct long-term studies about the effects of flossing treatment on the range of motion and performance (e.g., strength or jumping parameters) and its related mechanism (e.g., pain tolerance). There is weak evidence that flossing can be of value for pain relief in the treatment of certain diseases and for speeding up recovery after exercise. Moreover, there is weak evidence that flossing might have a superior conditioning (warm-up) effect compared to stretching when the goal is to improve the range of motion or certain aspects of muscle strength, while no such superior effect has been reported when compared to foam rolling.

## Introduction

**Figure 1:**
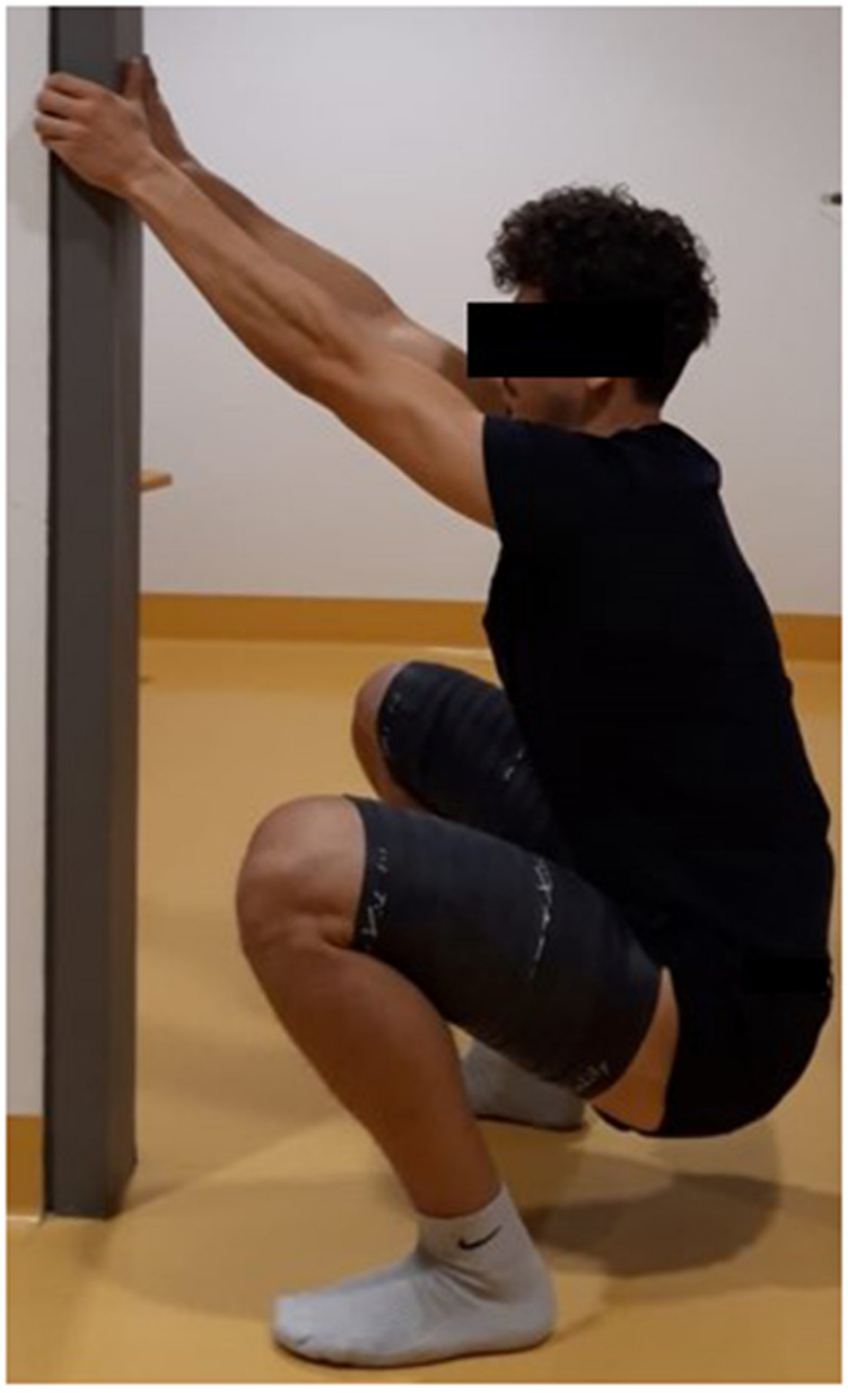
Typical example of a floss band treatment, with the floss band wrapped around the thigh. An additional movement during the floss band treatment (e.g., deep squat during thigh flossing) should enhance the efficacy.

Tissue flossing was first proposed by Starrett and Cordoza (2015), who suggested that flossing can increase the range of motion and/or performance (e.g., strength or jumping performance), speed up recovery, and decrease pain caused by various disease or injuries. One of the earliest peer-reviewed papers on the topic of floss band application (Driller and Overmayer, 2017) recommended wrapping the floss bland around a limb for 1 to 3 min and to overlap the floss band by 50% with regard to the previous wrap. Such a floss band application can be applied either on a joint and/or on soft tissue (see an example in Figure 1). In addition, it has been recommended that movements of the flossed joint or muscles are performed to end range of motion, to enhance the efficacy of the application (Starrett and Cordoza, 2015). During, for example, ankle or calf flossing, such a movement can be a plantarflexion movement to end range followed by a dorsiflexion movement to end range or just a standing wall push stretch (Konrad and Tilp, 2014), with the goal being to increase the dorsiflexion range of motion.

In general, there is a belief that flossing can stimulate the mechanoreceptors in the underlying fascial layers, leading to reperfusion of the compressed tissue (hence leading to enhanced blood flow), or causes fascial shearing, and consequently the fascia's sliding potential will be restored (Starrett and Cordoza, 2015; Stevenson et al., 2019). However, as of the time of writing, there was only some little scientific evidence on the responsible mechanisms behind the possible changes on the range of motion and performance parameters (e.g., strength or jump performance). Starrett and Cordoza (2015), e.g., suggested that possible mechanisms for increases in the range of motion could be related to fascial shearing or an increased reperfusion of blood following a vascular occlusion.

While there have been several review papers published about the effects of the more common techniques such as stretching or foam rolling on, for example, the range of motion (Behm et al., 2016; Wilke et al., 2020) or performance parameters (Behm and Wilke, 2019; Konrad et al., 2021), no such overview exists for the effects of flossing. To date, two reviews with similar specific research questions have been published, i.e., if flossing of the ankle or calf can increase the dorsiflexion range of motion (Kielur and Powden, 2020; Pisz et al., 2020) or jump performance (Pisz et al., 2020). Although these reviews investigated the range of motion of one joint (ankle), other effects of flossing, e.g., an increase in strength, increased recovery, or decrease in pain caused by various diseases or injuries, were not covered by these reviews. However, a review which covers the whole scope of flossing does not exist to date.

Since flossing is becoming more and more popular, the topic is now of great relevance not only for sports science but also for sports practice and therapy. Consequently, there is a requirement to summarize the available evidence and to identify knowledge gaps for future studies.

Therefore, the purpose of this scoping review is to provide an overview of the whole scope of flossing and to clarify the effects and the mechanisms of both a single floss band treatment and a repeated floss band treatment over several weeks on the range of motion, performance parameters (e.g., strength or jump performance), pain (caused by various diseases or injuries), injury, and disease, and also on recovery (e.g., delayed-onset muscle soreness, DOMS) in any kind of participant (e.g., athletes, patients). A further goal is to compare a floss band treatment with other treatments (e.g., stretching or foam rolling) and to clarify if different pressure levels for the applied floss band have an impact on various outcomes.

## Materials and Methods

**Figure 2:**
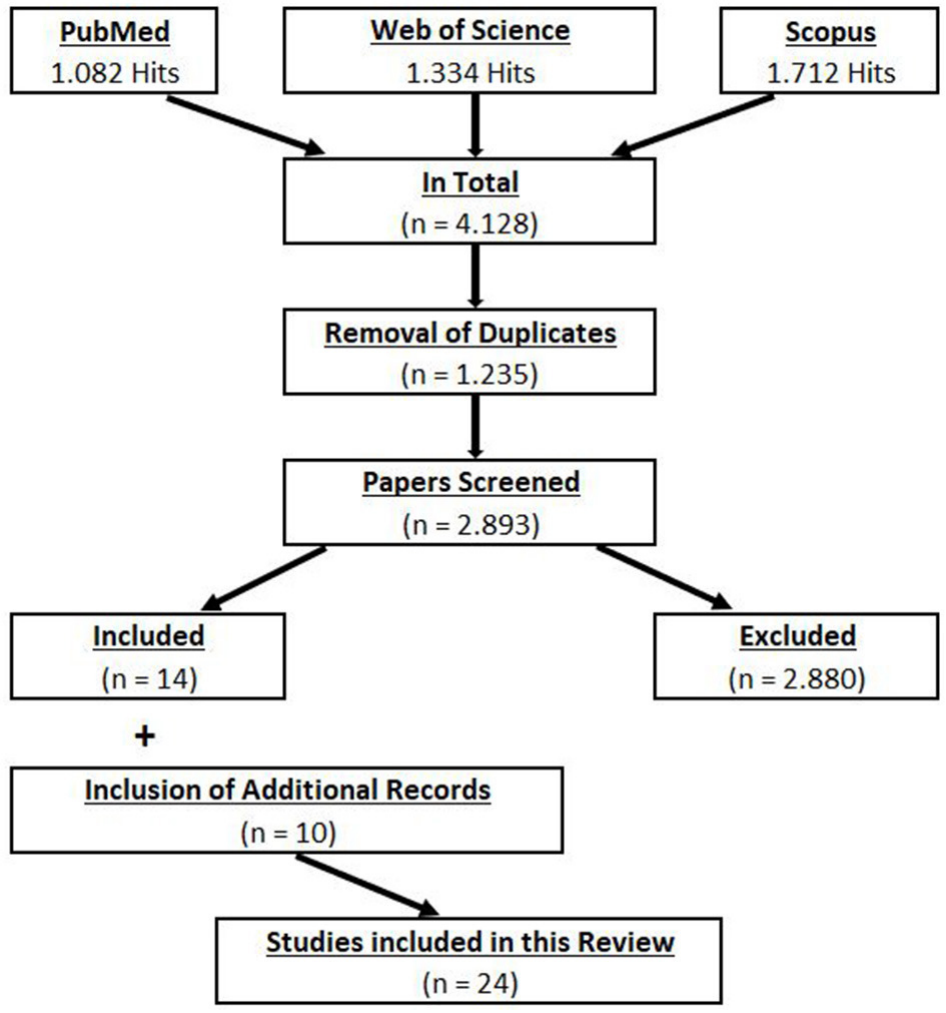
Flowchart of the systematic screening process (Preferred Reporting Items for Systematic Reviews and Meta-analyses).

This review is based on the suggestions made by Munn et al. (2018) with regard to scoping reviews. Thus, the aims of this review are to identify the available evidence and to identify knowledge gaps. To provide an overview of the whole scope of flossing, all kinds of published studies (original papers, case studies, pilot studies, and conference proceedings) were included in this scoping review, which is consistent with the suggestions of Munn et al. (2018). The electronic literature search was performed in three different databases (PubMed, Scopus, and Web of Science) using search terms/key words such as “flossing OR floss band OR vascular occlusion OR ischemic preconditioning AND sport^*^ OR performance OR rom OR recovery OR pain NOT dental NOT animal.” Since flossing is a relatively new technique, the search was restricted to the period from the year 2000 to November 23, 2020 (i.e., the date of extraction of all the literature from the databases). The inclusion criteria for this scoping review were any studies dealing with the effects of a floss band treatment on the range of motion, performance, recovery, or pain parameters in any population (e.g., patients, athletes) in English and German language. This process resulted in a total number of 4,128 studies being identified. After removing the duplicates (1,235), the remaining studies were screened independently by two researchers (AK and RM) by title (or if necessary by abstract) to identify the studies to be included in this review. Following this blind screening process, the researchers compared their findings and discussed possible mismatches. Overall, 2,893 studies were screened, but only 14 met the inclusion criteria and hence were included in this review. Moreover, through an additional search of the references and the citations (Scopus and Google Scholar) of the 14 papers already included, 10 more studies were identified and included. Therefore, in total, 24 papers were included in this scoping review. A detailed illustration of the search process is provided in Figure 2. Moreover, the characteristics of the included studies are presented in Table 1.

**Table 1:** Characteristics of the participants of all the included studies (*n* = 24) and a description of the floss band application.

**Study**	**Participants**	**Joint/tissue flossed**	**Floss band application**
**Single treatments**			
Cheatham et al. (2020)	30 (15 m/15 f) healthy, active adults; age: 25.43 ± 2.46 years	Thigh	Active movement: 30 s standing hip flexion + 30 s seated leg extension/flexion + 60 s bodyweight squats (∑ 2 min)
Driller and Overmayer (2017)	52 (29 m/23 f) recreational athletes; age: 20 ± 4 years	Ankle	Active movement: 20 reps of dorsi/plantar-flexions (full ROM) (Σ 2 min)
Driller et al. (2017)	69 (32 m/37 f) recreational athletes; age: 19 ± 2 years	Ankle	Active movement: 20 reps of dorsi/plantar-flexions (full ROM) (Σ 2 min)
Galis and Cooper (2020)	30 (16 m/14 f) healthy subjects; age (male): 21.5 ± 2.57 years, age (female): 20.79 ± 0.69 years	Calf	Active movement: dorsi/plantar-flexion (full ROM) for 2 min + 20 bodyweight squats
Marco et al. (2020)	5 male recreational athletes with patellofemoral pain syndrome; age: 22 ± 0.5 years	Knee	Active movement: 3 CMJ with the band on the painful knee (15 s between each Jump)
Gorny and Stöggl (2018)	42 (12 m/30 f) recreational athletes; age: 24.6 ± 4.3 years	Whole leg	Passive application: 2 times 3 × 2 min; first = post M2, second = post M3 (∑ 12 min within 60 min)
Kaneda et al. (2020b)	17 male healthy subjects; age: 23.2 ± 1.1 years	Hamstring	Active movement: two cycles of: manual twisting of the wrapped part (4 times) + 20 knee flexions/extensions−2 min rest in between (Σ ~4 min)
Kaneda et al. (2020a)	20 male recreational. athletes; age: 22.5 ± 1.0 years	Calf	Active movement: two cycles of: manual twisting of the wrapped part (4 times) + 20 plantar/dorsi-flexions−2 min rest in between (Σ ~4 min)
Kiefer et al. (2017)	60 subjects; age: 18–24 years	Shoulder	Active stretch: 5 × 30 s of “child's pose stretch” (∑ 2.5 min)
Konrad et al. (2020a)	16 male healthy subjects; age: 25.69 ± 4.1 years	Thigh	Active movement: 20 bodyweight deep squats within 2 min (Σ 2 min)
Mills et al. (2019)	14 male professional rugby players; age: 23.9 ± 2.7 years	Ankle	Active movement: 20 reps of dorsi/plantar-flexions (full ROM) (Σ 2 min)
Pakarklis and Šiupšinskas (2018)	26 (12 m/14 f) active athletes; age (male): 25.08 ± 4.32 years, age (female): 21.64 ± 4.24 years	Calf	Active movement: 20 reps of dorsi/plantar-flexions (full ROM) (Σ ~2 min)
Prill et al. (2019)	15 (8 m/7 f) healthy subjects; age: 21.9 ± 2.3 years	Biceps	Active movement: elbow-flexion/extension + internal/external rotation of the glenohumeral joint (∑ 3 min)
Plocker et al. (2015)	17 male athletes; age: Ø 20.7 years	Shoulder	Active movement: “shoulder prehabilitation exercises”
Ross and Kandassamy (2017)	10 (5 m/5 f) subjects; age: 23.8 ± 4.66 years	Calf	Active movement: deep squats and full ROM dorsiflexion (∑ 2.5 min)
Stevenson et al. (2019)	5 male recreational athletes; age: Ø 23.6 years	Ankle	Active movement: 20 reps of dorsi/plantar-flexion and circumduction + 10 squats + 15 eccentric heel raisers (∑~2 min)
Vogrin et al. (2021)	30 (12 m/18 f) recreational athletes; age: 23.0 ± 4.51 years	Ankle	Active movement: 3 times 2 min of slow dorsi/plantar-flexion (full ROM)−2 min rest in between (∑ 6 min)
Vogrin et al. (2020)	19 (14 m/5 f) recreational athletes; age: 23.8 ± 4.8 years	Thigh	Active movement: 3 times 2 min of slow knee flexion/extension (90° knee flexion to full knee extension)−2 min rest in between (∑ 6 min)
**Short- and long-term treatments**			
Bohlen et al. (2014)	5 (1 m/4 f) subjects; age: 20 ± 1 years	Calf	Total of 2 weeks: 1 session/day with active movement−2 × 10 bodyweight squats, 10 heel raises, 10 dorsi/plantar-flexions + passive ankle mobilization (∑ 14 flossing sessions)
Borda and Selhorst (2017)	1 female patient; age: 14	Ankle	Up to 9 months: 1 session/day with active movement−3 × 10 weight-bearing lunges (as a “flossing motion”) + Lacrosse ball massage
Cage et al. (2018)	1 male patient; age: 21	Wrist	Total of 6 weeks: 1 to 3 min with active movement−20 wrist joint rotations (clockwise and counterclockwise)
Carlson et al. (2019)	16 (4 m/12 f) adults; age: 18+	Ankle	Total of 4 weeks: 2 sessions/week with active movement−2 × 20 ankle pumps (∑ 8 flossing sessions)
Weber (2018)	1 male patient; age: 14	Knee	Total of 9 weeks: 3 sessions/week with active movement−10 bodyweight squats + 10 lunges (∑ 27 flossing sessions)
Wienke et al. (2020)	12 (6 m/6 f) patients; age: 48.0 ± 15.3 years (ranging from 21 to 74 years)	Shoulder	Total of 3 weeks: 5 flossing sessions within this time−3 times 2 min with active and passive motion of the shoulder joint (∑ 30 min)

*ROM, range of motion; CMJ, countermovement jump*.

The mean percentage changes (pre- to post-) and the 95% confidence intervals (CIs) of the range of motion and performance parameters are presented in the subsequent chapters. In addition, according to previous suggestions (Hopkins, 2004; Behm et al., 2016), we defined the magnitude of the calculated percentage mean changes of the range of motion and performance, i.e., we defined <0.5, 0.5 to <2, 2 to <5, 5 to <10, and >10% as trivial, small, moderate, large, and very large, respectively.

## The Impact of a Flossing Application on the Range of Motion

### Acute Effects

In total, 15 studies investigated the acute effects of a single floss band application on the range of motion of several joints. Eight studies applied the floss band on soft tissue (four thigh/four calf), and the other studies applied the floss band on the joints, namely, on the ankle (five studies) or on the upper body (two shoulder/one elbow). For these 15 studies, 29 range of motion measures were extracted since some studies analyzed various functions of the joint (e.g., plantar- and dorsiflexion at the ankle joint) (for more information, see Table 2).

**Table 2:** Summary of the results of the studies which investigated the acute effect of flossing on range of motion (before and after difference).

**Joint/tissue flossed**	**Study**	**Range of motion test applied**	**Change (pre–post)**	**Difference to controls**
Ankle	Driller and Overmayer, 2017	Plantarflexion	3.09%	1.86%
		Dorsiflexion	7.37%	6.29%
		Weight-bearing lunge test	16.51%	14.76%
	Driller et al., 2017	Weight-bearing lunge test	8.99%	8.99%
	Mills et al., 2019	Weight-bearing lunge test	4.04%	−0.08%
	Stevenson et al., 2019	Dorsiflexion	105%	74.23%
		Plantarflexion	6.30%	−7.77%
		Weight-bearing lunge test straight leg	20.20%	5.25%
		Weight-bearing lunge test bend leg	24.20%	14.44%
	Vogrin et al., 2021	Dorsiflexion	2.10%	1.65%
		Plantarflexion	1.90%	1.45%
Calf	Kaneda et al., 2020a	Dorsiflexion	32.90%	42.11%
	Pakarklis and Šiupšinskas, 2018	Dorsiflexion open kinematic chain	16.08%	1.00%
		Dorsiflexion closed kinematic chain	7.88%	5.58%
	Ross and Kandassamy, 2017	Dorsiflexion (left leg)	29.1%^a^	No control
		Dorsiflexion (right leg)	16.4%^a^	No control
	Galis and Cooper, 2020	Dorsiflexion (150 mmHg)	22.60%	25.24%
		Plantarflexion (150 mmHg)	2.60%	6.12%
		Dorsiflexion (200 mmHg)	12.88%	15.52%
		Plantarflexion (200 mmHg)	−8.20%	−4.68%
Thigh	Cheatham et al., 2020	Knee flexion	3.61%	No control
	Kaneda et al., 2020b	Straight leg test	13.36%	8.93%
		Passive knee extension test	4.50%	4.05%
	Konrad et al., 2020a	Modified Thomas test	−7.05%	−12.35%
	Vogrin et al., 2020	Straight leg test (low-pressure flossing)	1.40%	2.65%
		Straight leg test (high-pressure flossing)	0.50%	1.75%
Shoulder	Kiefer et al., 2017	Shoulder ROM	1.69%	−0.33%
	Plocker et al., 2015	Shoulder ROM (internal rotation)	nr	–
		Shoulder ROM (external rotation)	nr	–

*The green color indicates that a significant increase was found in the respective study, while the orange color indicates no significant change. A positive value in the column “Difference to controls” indicates a favorable effect of the flossing treatment to the control condition (and vice versa)*.

a *No significance level was reported*.

*nr, not reported; ROM, range of motion*.

### Results

The results of the individual studies show that 15 out of the 29 range of motion measures showed a significant increase (individual results of the studies as pre–post comparison) following the floss band application (see Table 2, green color). However, out of these 15 measures, in three measures, the same increase in range of motion was also found in the control group (no floss band applied, but a stretch or movement was performed) (Kiefer et al., 2017; Pakarklis and Šiupšinskas, 2018; Vogrin et al., 2021). In most of the included studies, the subjects were asked to perform a movement or a stretch during both the flossing condition and the control condition (see Table 1 for more information). Therefore, these findings indicate that a movement or stretch alone, without a floss band, can lead to a similar conditioning effect. Hence, no favorable effect of flossing was confirmed in these three studies compared to the control condition (i.e., stretching or movement without a floss band). This is in line with a meta-analysis about ankle joint flossing on the dorsiflexion range of motion (Kielur and Powden, 2020), where the authors reported significant increases in the before-to-after comparison, but there were no significant changes when the control groups were included in the meta-analysis (Kielur and Powden, 2020). However, other studies with control groups dealing with other joints than the ankle joint (which was exclusively explored by Kielur and Powden, 2020) have reported a positive effect on the range of motion from a floss band application compared to the control group (e.g., hamstring muscles in Kaneda et al., 2020b). Hence, muscle-specific effects can be assumed.

Moreover, 12 further measures showed no significant change in the range of motion following a floss band application (see Table 2, orange color). Please note that one study with two measures in total did not report the significance levels of their results (Ross and Kandassamy, 2017). In addition, it is of great interest that none of the 15 studies reported a significant decrease in the range of motion.

Taking the significant and non-significant findings together, out of the 29 measures, in 27 measures, the percentage changes of the before-to-after comparison were reported. Concerning ankle flossing, 11 measures from five studies were extracted. Out of these 11 measures, seven were significantly increased. By considering just the main measure of each study on ankle flossing (weight bearing lunge test in Driller and Overmayer, 2017; Driller et al., 2017; Mills et al., 2019; dorsiflexion range of motion in Vogrin et al., 2021), the mean change was a very large increase in the dorsiflexion range of motion of 11.17% (95% CI, 4.25 to 18.63%). With regard to calf flossing, nine measures from four studies are presented in Table 2. Out of the nine measures on calf flossing, four were significantly increased. By considering just the main measure of each study on calf flossing (dorsiflexion range of motion in Ross and Kandassamy, 2017; Pakarklis and Šiupšinskas, 2018; Galis and Cooper, 2020; Kaneda et al., 2020a), the mean change was a very large increase in the dorsiflexion range of motion of 19.95% (95% CI, 11.56 to 28.78%). Moreover, four studies performed thigh flossing including six measures in total. Three out of the six measures on thigh flossing were significantly increased. One study showed a significant increase of 3.61% in the knee flexion range of motion (Cheatham et al., 2020), while another study showed a non-significant decrease (−7.05%) in the hip flexion range of motion (Konrad et al., 2020a) following thigh flossing. Considering the two studies which investigated the knee extension range of motion with a straight leg test following thigh flossing (Kaneda et al., 2020b; Vogrin et al., 2020), a mean change of a large increase in the knee extension range of motion of 7.38% (95% CI, 1.40 to 13.36%) was shown.

A further meta-analysis underlined these findings since they reported an increase in the weight-bearing lunge test results (indication for the dorsiflexion range of motion) following both ankle and calf flossing (Pisz et al., 2020).

### Effect Sizes

According to the provided evidence, it is likely that a floss band application can increase the range of motion of a joint. However, the average effect size of the included studies is 0.398 (ranging from 0.01 to 0.7), indicating a small to moderate magnitude of change (Cohen, 1988). Thus, caution has to be taken to not overemphasize these findings. Moreover, it can be recommended that future studies should assume a small to moderate effect for their *a priori* sample size calculation if the range of motion is the main variable so as to not underpower the sample.

### Possible Mechanism

Only a handful of the selected studies investigated possible mechanisms behind such increases in the range of motion following a floss band treatment. With regard to a single stretching exercise, a possible mechanism for the changes in the range of motion is changes in soft tissue (e.g., muscle stiffness; Konrad and Tilp, 2020) and/or changes in the perception of stretching or pain (Magnusson et al., 1996). Kaneda et al. (2020b) reported that, following a floss band application on the thigh, the passive torque at end range of motion was significantly higher in the hamstring muscle. This would indicate changes in the perception of stretch or pain. Although not significant, they also reported lower passive torque values from −90° (−43.8%) to −10° (−16.7%) (assessed during a passive movement) following the floss band application, which would likely indicate an additional decrease in muscle (and/or tendon) stiffness. However, muscle and/or tendon stiffness was not assessed by Kaneda et al. (2020b). Nevertheless, in a further study by these same authors (Kaneda et al., 2020a), they again found an increase in the range of motion and passive torque at end range of motion following a floss band treatment. The passive torque values at 20° dorsiflexion (+0.9%) following flossing were similar in the pre- and post-measurements, indicating no change in the myotendinous tissue (e.g., stiffness). This was underlined by the fact that they found no changes in muscle stiffness following the floss band treatment. Controversially, the static stretching group (they compared static stretching with flossing) in this study showed a decrease in muscle stiffness and no change in stretch tolerance (Kaneda et al., 2020a). This indicates different mechanisms for the changes in the range of motion when comparing flossing and stretching. Moreover, in the study of Vogrin et al. (2021), the subjects flossed the ankle joint, and the authors reported an increase in the range of motion but no change in muscle stiffness. However, in this study, no torque angle curves or passive torque data at the end range of motion were reported. Thus, from the current evidence, it can be concluded that flossing likely increases stretch tolerance. Moreover, it is likely that flossing of the calf or ankle does not reduce muscle stiffness (of the calf muscles), while this may not be automatically true for other muscles (e.g., see the torque angle curve of the hamstrings in Kaneda et al., 2020a). Such different effects between muscle groups in neurological (e.g., stretch perception) or mechanical (e.g., muscle stiffness) changes have also been reported following other compression treatments (e.g., foam rolling). For example, Baumgart et al. (2019) reported a decrease in muscle stiffness following a single foam rolling session in the rectus femoris muscle, but not in the gastrocnemius muscle. Thus, it cannot be excluded that a floss band treatment of different muscles leads to different outcomes with regard to, e.g., range of motion or their mechanism (e.g., changes in muscle stiffness). A further mechanism for a possible increase in the range of motion following flossing might be found in thixotropic effects. This effect has also been suggested in foam rolling (Behm and Wilke, 2019), stretching (Behm, 2018, p. 48), and in massage (Konrad et al., 2020b). Similar to foam rolling, a flossing treatment causes pressure on the treated muscle, skin, and fascia. Hence, according to this theory, this could have an impact on fluid viscosity and would lead to less resistance to movement (Behm, 2018, p. 48; Behm and Wilke, 2019).

### Long-Term Effects

In total, only two studies investigated the long-term effects of a repeated floss band application on the range of motion of different joints. Wienke et al. (2020) investigated the effects of a flossing treatment in 12 patients with shoulder pain. In addition to the conventional therapy, the subjects received shoulder flossing treatment twice a week, and the range of motion of the shoulder was assessed before and after the treatment over 3 weeks. Although not statistically significant, an increase in shoulder range of motion was reported in the anteflexion range of motion (+7.6%), the abduction range of motion (+18.3%), and the external rotation range of motion (+3.8%). It is possible that the study was underpowered to detect clinically relevant changes. Moreover, in a further study, two treatments were compared, namely, an ankle flossing treatment and an instrumented soft tissue mobilization of the ankle (Carlson et al., 2019). These treatments were performed two times weekly over a time period of 4 weeks in 16 healthy subjects. The statistical analysis indicated an increase in the dorsiflexion range of motion in both groups, but no absolute or percentage changes were presented.

Since studies of the long-term effects of flossing have been diverse in terms of the population (patients vs. healthy participants) and the treated joints (ankle *vs*. shoulder) and due to the fact that the sample sizes have been rather small, no general conclusion should be made.

### Conclusions on the Effects of Flossing on the Range of Motion

According to the data that we extracted in this scoping review, it can be assumed that both joint flossing and soft tissue flossing have a positive impact on the range of motion, but with only a small to moderate magnitude of change. This might be explained by the fact that the control condition (movement or stretching alone, without a floss band treatment) induced similar changes in the range of motion in some (e.g., Vogrin et al., 2021) but not all studies (e.g., Kaneda et al., 2020a). Moreover, a possible mechanism for an increase in the range of motion following a single flossing application is likely related to increased stretch tolerance (Kaneda et al., 2020b) rather than changes in the stiffness of the myotendinous tissue (Kaneda et al., 2020a; Vogrin et al., 2021). However, more research is needed to explore further possible mechanisms which can be related to a possible increase in the range of motion following a flossing treatment. In addition, more studies are needed to determine the effects of a long-term flossing treatment of various populations (e.g., athletes, patients) on the range of motion of the various joints.

## The Impact of a Flossing Application on Performance

### Acute Effects

In total, 11 studies investigated the acute effects of a single floss band application on performance parameters such as countermovement jump performance or torque and power measurements on isokinetic dynamometers. Six studies applied the floss band on soft tissue (three thigh/three calf), and the other studies applied the floss band on the ankle joint (three studies), knee joint (one study), and shoulder joint (one study). Out of these 11 studies, 44 performance measures were extracted. Twenty-six measures were strength, torque, or power measures or rate of force development (RFD) measurements on an isokinetic dynamometer. Moreover, 10 measures were related to countermovement performance (e.g., height, velocity), seven measures were related to sprint performance (e.g., 5 to 20 m sprints), and one measure was related to balance performance (see Table 3 for more details).

**Table 3:** Summary of the results of the studies which investigated the acute effect of flossing on performance parameters.

**Joint/tissue flossed**	**Study**	**Performance test applied**	**Change (pre–post)**	**Difference to controls**
Ankle	Driller and Overmayer, 2017	CMJ height	17.40%	9.07%
		CMJ velocity	8%	6.45%
	Driller et al., 2017	CMJ force	2.30%	3.82%
		5 m sprint	−0.90%	0.00%
		10 m sprint	0%	2.04%
		15 m sprint	1.50%	2.22%
	Mills et al., 2019	CMJ force	1.30%	3.06%
		5 m sprint	−2.00%	−2.00%
		10 m sprint	0.60%	1.17%
		15 m sprint	0.00%	0.00%
		20 m sprint	−0.3%	−0.30%
Knee	Marco et al., 2020	CMJ height	11.10%	8.20%
		Time in the air	5.40%	2.84%
		CMJ velocity	6.00%	3.60%
		CMJ power	13.90%	10.58%
		CMJ force	8.10%	5.55%
Shoulder	Plocker et al., 2015	Upper extremity power	nr	–
Calf	Galis and Cooper, 2020	Torque dorsiflexion (150 mmHg)	7.87%	13.82%
		Torque plantarflexion (150 mmHg)	0.96%	0.25%
		Power dorsiflexion (150 mmHg)	12.16%	15.05%
		Power plantarflexion (150 mmHg)	0.07%	−4.42%
		Torque dorsiflexion (200 mmHg)	−1.88%	4.06%
		Torque plantarflexion (200 mmHg)	−5.63%	−6.34%
		Power dorsiflexion (200 mmHg)	−5.67%	−2.78%
		Power plantarflexion (200 mmHg)	−4.76%	−9.25%
	Kaneda et al., 2020a	MVC plantar flexors	0.00%	−8.33%
		RFD 0–50 ms	21.10%	15.04%
		RFD 0–100 ms	11.90%	0.79%
		RFD 0–150 ms	5.10%	−6.66%
		RFD 0–200 ms	6.10%	−0.35%
	Pakarklis and Šiupšinskas, 2018	Leg dynamic balance anterior direction	1.90%	1.81%
Thigh	Kaneda et al., 2020b	MVC knee flexors	3.90%	11.03%
		RFD 0–50 ms	−3.60%	7.51%
		RFD 0–100 ms	−6.70%	4.01%
		RFD 0–150 ms	−3.70%	8.30%
		RFD 0–200 ms	0.00%	13.64%
		Maximum eccentric knee extension	13.80%	10.71%
		Maximum eccentric knee flexion	8.30%	12.40%
	Konrad et al., 2020a	MVC knee extensors	5.62%	6.51%
		CMJ height	−1.40%	−0.02%
	Vogrin et al., 2020	MVC knee extensors (low-pressure flossing)	5.80%	5.01%
		MVC knee flexors (low-pressure flossing)	2.10%	0.37%
		MVC knee extensors (high-pressure flossing)	2.60%	1.81%
		MVC knee flexors (high-pressure flossing)	3.80%	2.07%

*The green color indicates that a significant increase was found in the respective study, while the orange color indicates no significant change. A positive value in the column “Difference to controls” indicates a favorable effect of the flossing treatment to the control condition (and vice versa)*.

*CMJ, countermovement jump; MVC, maximum voluntary contraction; RFD, rate of force development; nr, not reported*.

### Results

The results of the individual studies show that 11 out of the 44 performance measures showed a significant improvement (i.e., individual results of the studies as a pre–post comparison) following the floss band application (see Table 3, in green color). Out of these 11 measures, there were seven jump parameters, and four parameters were isokinetic parameters [RFD and maximum voluntary contraction (MVC)]. The remaining 33 parameters (22 measures were isokinetic parameters, three were countermovement performance parameters, seven were sprint performance parameters, and one was a balance parameter) showed no significant change in performance (see Table 3, in orange color). Thus, as with the range of motion, no (significant) negative effect of a floss band treatment has been reported so far. Out of these 33 measures with insignificant findings, it has to be noted that the effect size of two measures in the study of Driller et al. (2017) (CMJ force and 15 m sprint performance), one measure in Mills et al. (2019) (CMJ force), and one measure in Galis and Cooper (2020) (dorsiflexion power) indicated a favorable effect of the flossing treatment compared to a control condition (movement without a floss band).

Taking the significant and non-significant findings together out of the 44 measures, in 43 measures, percentage changes were extracted. Concerning ankle flossing, 11 measures from three studies were extracted. Only two out of the 11 measures on ankle flossing showed a significant increase in performance. One study reported a significant increase in CMJ height following ankle flossing (Driller and Overmayer, 2017). Two studies (Driller et al., 2017; Mills et al., 2019) showed trivial to small changes in sprinting times following ankle flossing [5 m: −1.45% (95% CI, −2.00 to −0.90%); 10 m: 0.3% (95% CI, 0.00 to 0.60%); 15 m: 0.75% (95% CI, 0.00 to 1.50%)]. With regard to calf flossing, 14 measures out of three studies were extracted. Only one out of the six measures demonstrated a significant improvement following calf flossing on performance (RFD 0–50 ms in Kaneda et al., 2020a). While plantar flexor isometric strength (Galis and Cooper, 2020; Kaneda et al., 2020a) and balance (Pakarklis and Šiupšinskas, 2018) seem to be unchanged following calf flossing, RFD in various time frames (e.g., 0–50 ms) showed an increase ranging from 5.10 to 21.10% (Kaneda et al., 2020a). Knee flossing was only applied in one study (five measures) with all parameters (related to jumping) significantly improved when comparing before and after values (Marco et al., 2020). Thigh flossing was performed in three studies with a total of 13 measures. However, only three out of the 13 measures showed a significant increase following the flossing treatment. One study showed a non-significant decrease in CMJ height (−1.40%; Konrad et al., 2020a), and another study showed non-significant decreases in various time frames of RFD (e.g., 0–50 ms) ranging from −6.70 to 0.00% (Kaneda et al., 2020b). However, concerning MVC of both knee extensors and knee flexors, a moderate average increase was shown. The two measures of the knee extensor MVC (out of two studies) showed an average increase of 5.71% (95% CI, 5.62 to 5.80%). It has to be mentioned here that both studies reported these increases to be significant (Konrad et al., 2020a; Vogrin et al., 2020). Although the individual study results were shown to be not significant, the knee flexor MVC following thigh flossing, taken out of two studies (Kaneda et al., 2020b; Vogrin et al., 2020), showed an average increase of 3.00% (95% CI, 2.10 to 3.90%).

In summary, there is small evidence that joint flossing (knee and ankle) may increase jump height, while sprint performance seems to be unaffected following ankle flossing. Moreover, thigh flossing seems to be a proper tool to increase the MVC of both the knee extensors and the knee flexors.

### Effect Sizes

According to the included studies about the acute effects of a floss band treatment, an increase in performance might indeed be possible. However, as in the findings for range of motion, these outcomes are not supported by high effect sizes. The effect size reported by the studies is, on average, 0.244 (ranging from 0 to 0.77), indicating a small to moderate magnitude of change (Cohen, 1988).

### Possible Mechanism

A suggested mechanism by Driller et al. (2017) underpinning a possible increase in performance could be a hormonal response related to the flossing treatment. With regard to other occlusion methods, enhanced growth hormone and sympathetic hormone (norepinephrine) levels after the release of the compression were reported by Takarada et al. (2006). This might also be true after the compression release of a floss band and could be the mechanism responsible for the increase in performance reported in some studies. Moreover, Konrad et al. (2020a) suggested that, among the other responses related to an increase of sympathetic outflow, there may be a facilitation of the short-latency stretch reflex (Hjortskov et al., 2005). It has long been known that afferents from muscle spindles contribute in various ways to different voluntary muscle contractions (Macefield et al., 1991), and thus an increase in spinal excitability can provoke an increase in performance. This goes in line with the results of Kaneda et al. (2020a), who reported a higher RFD (0–50 ms) of the plantar flexors and increased muscle activity of the gastrocnemius lateralis muscles following the floss band treatment, indicating neurological adaptations. However, enhanced muscle activation was not reported in a further study by Kaneda et al. (2020b) about the effects on the hamstring muscles and the study of Konrad et al. (2020a) about the effects on the quadriceps muscle, although both studies reported increases in performance. As in a previous work (Konrad et al., 2020a), Vogrin et al. (2020) reported increased knee extensor MVC. Furthermore, Vogrin et al. (2020) reported a reduction in contraction time in the rectus femoris muscle (but not in the vastus medialis or biceps femoris) assessed with tensiomyography, which likely results in neuromuscular potentiation. Thus, the authors concluded that the increase in knee extensor MVC could likely be explained by improved neuromuscular function.

### Short-Term Effects

Only one study in terms of a conference proceeding (Bohlen et al., 2014) investigated the short-term effects of a floss band treatment on performance parameters. The five participants in this study applied the floss band daily for a period of 14 days, proximal and distal to the patellar (on the experimental leg only), and performed bilateral resistive exercises (i.e., air squats, heel raises, active dorsiflexion). The other leg served as a control. Dorsiflexion peak torque showed an increase (+22%; *P* = 0.06) following the 14 day intervention period. Although no further information about the exact wrapping technique or pressure was reported, the findings of Bohlen et al. (2014) are an important first step in further research questions related to this topic. To date, no study investigated the mechanism behind the possible changes in performance following a floss band treatment repeated for several weeks. Although speculative, the most likely mechanism is neurological adaptations rather than any mechano-morphological changes.

### Conclusions on the Effects of Flossing on Performance

According to the involved studies, it can be concluded that a single floss band treatment seems to have no detrimental effects on performance. In contrast, there is small evidence that joint flossing (knee and ankle) may increase jump height, and thigh flossing might have a positive impact on knee extensor and knee flexor MVC. However, the reported effect sizes are rather small (mean, 0.244; ranging from 0 to 0.77). The possible mechanisms for such an increase in performance are increased muscle activity and improved neuromuscular function (Kaneda et al., 2020a; Vogrin et al., 2020). We found no reports about the effects of a single floss band application on the performance (e.g., throwing) of the upper body. The possible mechanisms for the change in performance need to be further investigated (e.g., changes in growth hormone). Moreover, there is also a need to investigate the long-term effects of a flossing treatment on any joint.

## The Impact of Flossing on Disease, Pain, and Injury

In total, five studies investigated the effects of a flossing treatment on disease, pain, or injury.

A case study (Borda and Selhorst, 2017) about Achilles tendinopathy reported that only two sessions of an ankle floss band treatment, plus a lacrosse ball massage, were effective in reducing pain and increasing the Lower Extremity Functional Scale score of a 14 year-old female patient. It should also be noted that 6 weeks of prior traditional physiotherapy failed to enhance the situation of this patient. However, as this case study was not a controlled study, it cannot be established if the improvement in pain was due to the floss band treatment, the lacrosse ball massage (or the combination), or even a placebo effect. A further case study reported a significant improvement in Kienböck's disease (in the visual analog scale and wrist/hand disability index) of a 21 year-old male basketball player following a 6 week period of wrist flossing (Cage et al., 2018). With regard to Osgood–Schlatter's disease, an additional case study reported improvement in both pain and muscle function following a 9 week floss band intervention at the knee joint of a 14 year-old male soccer player (Weber, 2018). Although these results are very important indications for future studies and for therapists, due to the lack of a control intervention and the fact that these studies were case studies, it cannot be excluded that other factors were responsible for the improvements seen in these studies.

Moreover, a further controlled study (Wienke et al., 2020) investigated the effects of a flossing treatment in 12 patients (six intervention/six control) with shoulder pain. The subjects of the intervention group received shoulder flossing twice a week, whereas the control group received a “sham” flossing treatment. Not only the pain level of the shoulder but also the shoulder range of motion was assessed before and after the treatment over 3 weeks. No significant change in pain of the shoulder was reported, though it is possible that the study was underpowered to detect clinically relevant changes. Furthermore, a pilot study (Marco et al., 2020) with five young male athletes who suffered from knee pain reported significant improvements in both pain (visual analog scale) and jump performance following a single floss band treatment on the knee joint.

Since there is a lack of randomized controlled trials and studies with sufficient power, the generalizability of the findings is highly unclear to date. Although only case studies, studies with a relatively small sample size, and studies of various diseases have been reported here, there are common reports of a likely reduction in pain and an improvement in function following a floss band treatment. To date, no coherent theory about the possible mechanism on the effects of flossing on disease, pain, and injury exists. Furthermore, more evidence obtained with randomized controlled trials and a high sample size is needed to investigate the effects of a floss band treatment on pain and muscle function in various diseases.

## The Impact of Flossing on Recovery from Delayed-Onset Muscle Soreness

### Results

Only two studies investigated the effects of a flossing treatment on DOMS. Prill et al. (2019) investigated the effects of a single flossing treatment of the upper arm following exercise-induced muscle fatigue with different modes of contraction (concentric, eccentric, and isometric) in 17 participants on a visual analog scale. The participants reported significantly reduced DOMS on the measured time points (24 and 48 h post-exercise) compared to the control condition (no flossing). A further study (Gorny and Stöggl, 2018) investigated the effects of a flossing treatment of the leg muscles following leg-press-induced DOMS with the Likert muscle scale in 42 participants. No difference in Likert muscle scale was found 12, 24, 36, 48, 60, and 72 h post-exercise between the intervention group (*N* = 21) and the control group (*N* = 21; no flossing). Thus, the authors concluded that a flossing treatment cannot induce a faster recovery rate or reduce DOMS. A possible difference between these two reports might be found in the floss band application. In the study of Prill et al. (2019), the subjects had to move their arm in several directions (flexion, extension, internal/external rotation), but in the study of Gorny and Stöggl (2018), the subjects performed no movement during the application. Thus, it can be speculated that, due to the additional movement in the study of Prill et al. (2019), a more pronounced blood flow was induced after the compression release, compared to the study of Gorny and Stöggl (2018). Future studies should test the effect of movement during a floss band treatment.

Moreover, neither study measured the range of motion or performance changes following the exercise-induced DOMS. Since both the visual analog scale and Likert muscle scale are subjective measures, more objective measures like movement biomechanics (e.g., in running as reported in Paquette et al., 2017) might have led to a clearer picture if flossing can indeed speed up recovery.

### Possible Mechanism

To date, a possible mechanism for a decrease in DOMS remains hypothetical. Prill et al. (2019) and Gorny and Stöggl (2018) speculated in their discussion that compression therapy can reduce inflammation in the muscles when the floss band is applied immediately after the exercise-induced microstructural muscle damage. This damage is mainly caused by mechanical strain (e.g., due to training), which can induce disruption of the sarcomeres, causing a development of edema, which can, in turn, induce pain by increasing the sensitivity of the nociceptors (Brown et al., 2017). As with compression garments (Brown et al., 2017), it can be assumed that reduced intracellular osmotic pressure caused by the floss band application will lead to a decrease in the sensitivity of the nociceptors and hence to reduced DOMS. However, this assumption is mainly based on the effects of other compression garments (e.g., lower limb compression), which, on the one hand, do not apply that much pressure to the skin (~15 mmHg; Brown et al., 2017) compared to a floss band treatment (e.g., ~180 mmHg; Driller et al., 2017) but, on the other hand, are worn for a longer time period (e.g., 12 h; Jakeman et al., 2010) compared to the relatively short time period of a floss band application (1 to 3 min; Driller et al., 2017). Hence, there is a need for further studies to explore the specific effects of a floss band application on DOMS and recovery and also to explain which mechanism might be responsible for a possible change in DOMS and recovery.

## The Impact of the Pressure Level of the Floss Band

### How to Assess the Pressure

Out of the 24 studies, only 10 studies monitored and reported the pressure of the floss band applied to the skin. This was mainly done with a Kikuhime pressure measurement device (e.g., Driller and Overmayer, 2017; Vogrin et al., 2020) or an adapted sphygmomanometer (e.g., Galis and Cooper, 2020; Konrad et al., 2020a). In the studies of Driller and Overmayer (2017), Driller et al. (2017), and Mills et al. (2019), the Kikuhime pressure measurement device was shown to be both reliable (CV = 4.9%) and valid (ICC = 0.99, CV = 1.1%).

### Variability of the Pressure Within and Between the Studies

The average pressure reported by the 10 studies was 167.3 ± 24.6 mmHg, with a range of 120–210 mmHg. This variety of pressure could have had an impact on the results found between and also within the studies. Comparisons between the studies showed that Kaneda et al. (2020b) applied an average pressure on the thigh of 134 ± 10.2 mmHg, while Konrad et al. (2020a) applied 20 mmHg more pressure on average (154.3 ± 13.3 mmHg) on the same tissue. Since it is not possible for a therapist (or athlete/patient) to wrap the floss band with exactly the same pressure, the studies also reported standard deviations of the pressures applied between 3 and 38 mmHg. It can be assumed that the differences in the pressure where the floss band is applied can have an impact on the results both between and within studies.

### Low Pressure vs. High Pressure

That varying pressure can lead to different results is underlined by the findings of Vogrin et al. (2020) and Galis and Cooper (2020), who compared low-pressure with high-pressure floss band application. While Vogrin et al. (2020) defined low-pressure application based on the circumference of the thigh as 100–140 mmHg and high-pressure application as 150–210 mmHg, Galis and Cooper (2020) reported 150 and 200 mmHg as being low pressure and high pressure, respectively. Both studies reported favorable results with the low-pressure floss band applications in terms of the range of motion and strength parameters (Galis and Cooper, 2020; Vogrin et al., 2020). Galis and Cooper (2020) even reported some adverse effects in the high-pressure group in both the range of motion and strength outcome and therefore concluded that tighter does not automatically mean better.

Moreover, compression garments are used by athletes in the belief that they can speed up recovery. Unlike flossing, compression garments can be worn for several hours since the pressure is ~10-fold lower than that in a typical floss band application. A meta-analysis of compression garments (Brown et al., 2017) did not find a significant difference between the so-called high-pressure (≥15 mmHg) and low-pressure (<15 mmHg) garments in recovery from exercise. However, there was a trend (*P* = 0.06) in that the higher-pressure garments showed poorer recovery than the lower-pressure garments. Thus, it can be speculated that both floss bands and compression garments should be applied with lower pressure (<150 mmHg for floss band treatments and <15 mmHg for compression garments) to ensure a better outcome. However, more research is needed to investigate the effects of various pressures of the floss band application on different output parameters (e.g., recovery, range of motion, and performance).

### Possible Other Approaches to Measure the Pressure

To conduct a more practical approach to measure the pressure, it might be worth investigating the pressure levels based on the participants' subjective feeling (e.g., pleasant to painful pressure), which is an accepted method in foam rolling (Nakamura et al., 2021) and stretching studies (Konrad and Tilp, 2020). Moreover, a further approach by taking the stretch of the band to a given extent (e.g., 25 and 50%) where the estimated force has already been assessed (Cheatham and Baker, 2019) would likely make future studies more comparable and should help athletes, therapists, and patients better understand how to apply a floss band, based on the study findings, and hence to get the most benefit out of it.

### Harmful Effects Due to the Pressure of the Floss Band

Caution should be taken with too much pressure applied by the floss bands. Galis and Cooper (2020) reported some adverse effects in the high-pressure group (200 mmHg) compared to the low- pressure group (150 mmHg) in both the range of motion and strength outcome and therefore concluded that tighter does not automatically mean better. Even worse, one study reported harmful effects, e.g., violent pain, changes in the color of the skin, hematoma, or numbness, in participants who received a floss band treatment on the shoulder (Wienke et al., 2020). Thus, it is strongly suggested not only in future studies but also in sport practice to monitor the possible harmful effects due to a floss band application.

## Flossing Compared With Other Treatments

To evaluate if flossing can have a positive effect for athletes or patients, there is a need to compare different warm-up strategies and therapy modalities with a floss band treatment. It is known from the literature that a single stretching exercise can increase the range of motion of a joint (e.g., Konrad et al., 2017b); however, if the duration of the stretch exceeds >60 s, it is very likely that impairments in strength parameters will occur (Behm et al., 2016). Two studies from one research group investigated the differences between a floss band treatment and dynamic stretching (Kaneda et al., 2020b) and static stretching (Kaneda et al., 2020a). Compared to dynamic stretching, Kaneda et al. (2020b) reported a more pronounced effect with a floss band treatment in the increase in hip range of motion and one strength parameter (maximal eccentric knee extension torque). With regard to static stretching and the comparison with a floss band treatment, Kaneda et al. (2020a) reported that RFD was more pronounced in the flossing group than the static stretching group (calf muscles). Interestingly, while the mechanism for the increase in the range of motion following static stretching was considered to be reduced muscle stiffness, the floss band treatment showed neurological adaptations as suggested by the observed increase in stretch tolerance (i.e., higher passive torque at end range of motion). Kaneda and colleagues concluded in both studies (Kaneda et al., 2020a,b) that flossing should be applied as a warm-up treatment rather than as a stretching exercise.

A further frequently investigated warm-up modality, besides stretching, is foam rolling, in terms of possible benefits in the range of motion (Nakamura et al., 2021), strength (Reiner et al., 2021), or recovery (Nakamura et al., 2020). Cheatham et al. (2020) compared a flossing treatment with a single session of foam rolling and also with instrument-assisted soft tissue mobilization (a kind of massage). All three modalities showed an increase in the range of motion, but there was no difference found between the modalities. In addition, Carlson et al. (2019) reported the same findings when comparing instrument-assisted soft tissue mobilization of the ankle with ankle flossing.

To date, there is not much evidence about the possible benefits of flossing compared to other treatments. When compared to stretching, flossing showed a superior conditioning (warm-up) effect in a few parameters (Kaneda et al., 2020a: RFD; Kaneda et al., 2020b: range of motion, maximal eccentric knee extension), but in most parameters (Kaneda et al., 2020a: range of motion, MVC; Kaneda et al., 2020b: maximal eccentric knee flexion; MVC; RFD), no significant difference was reported. Moreover, the mechanism which is responsible for the changes in the range of motion or performance following flossing is likely different to that in stretching. While the changes in the range of motion following stretching are likely related to reduced muscle stiffness (Konrad et al., 2019; Kaneda et al., 2020a) and/or increased stretch tolerance (e.g., Magnusson et al., 1996; Konrad et al., 2017a), only the latter seems to contribute to the increase in the range of motion after flossing (Kaneda et al., 2020a). No differences in the effects on the range of motion have been detected between flossing and foam rolling and instrument-assisted soft tissue mobilization. However, more research is needed to compare the long-term effects of the different techniques (flossing vs. stretching) or even to investigate the combined effects (e.g., flossing and stretching combined).

## Discussion and Conclusion

This scoping review has provided an overview of the application of a floss band and summarizes the existing evidence on the effect of a floss band treatment on the range of motion, performance, recovery, and pain parameters. Moreover, the different pressure levels which can be applied and the relationship with other treatments (e.g., stretching) were discussed. In general, there is evidence that a floss band treatment applied either on the joint or the soft tissue is able to increase the range of motion of the related joint. Moreover, joint flossing may additionally increase jump height, and flossing applied on the thigh can positively affect isometric strength. However, these findings showed only small to moderate magnitudes of change in the included studies. Although not yet clearly understood, a possible mechanism for such changes in the range of motion is likely due to increased stretch tolerance rather than changes in the mechanical parameters of the muscle (e.g., stiffness). All in all, there is a need to conduct long-term studies about the effects of flossing treatments on various parameters like the range of motion or performance and their mechanism (e.g., pain tolerance). Moreover, some case studies and studies with small sample sizes reported a reduction in pain (caused by various diseases or injuries) following a flossing treatment. However, more evidence and controlled studies are necessary to understand the effects of a flossing treatment or intervention (e.g., for several weeks) on pain and injury. Only two studies reported conflicting results about flossing and its effect on recovery. In terms of the pressure of the applied floss band, it can be recommended to apply less pressure (<150 mmHg), rather than more, to avoid a possible adverse effect or even a harmful effect. There is weak evidence that flossing might have a superior conditioning (warm-up) effect in some parameters, compared to stretching, when the goal is to enhance the range of motion or strength parameters. However, no such superior effect was reported when compared to foam rolling or instrument-assisted soft tissue mobilization.

Since to date a lot of theories and only poor evidence exists on the topic of flossing, we strongly encourage performing further prospective studies about the acute and long-term impact on the healthy and impaired musculoskeletal system and the underlying mechanisms.

## Author Contributions

AK and RM collaborated in the literature review and in producing the figures and tables. AK, RM, and MN collaborated in writing the manuscript. All the authors contributed to the article and approved the submitted version.

## Conflict of Interest

The authors declare that the research was conducted in the absence of any commercial or financial relationships that could be construed as a potential conflict of interest.
